# Spatiotemporal Transcriptomics Reveals That a ZF‐HD–*GmCWINV4* Module Controls Carbon Partitioning and Seed Size in Soybean

**DOI:** 10.1111/pbi.70776

**Published:** 2026-10-28

**Authors:** Di Wu, Yiran Ding, Yang Tao, Xiaoyi Fu, Bing Han, Juan Song, Tianquan Yang, Tingshuang Yi, Wei Xu

**Affiliations:** ^1^ Germplasm Bank of Wild Species, Yunnan Key Laboratory of Crop Wild Relatives Omics Kunming Institute of Botany, Chinese Academy of Sciences Kunming Yunnan China; ^2^ University of Chinese Academy of Sciences Beijing China

**Keywords:** *GmCWINV4*, GmZF‐HD, seed size, soybean seed, spatiotemporal transcriptomics, transcriptional regulation

## Abstract

Seed size and oil content are key determinants of agronomic productivity in soybean ( *Glycine max* ). Although the ultrastructure and cellular composition of soybean seeds have been well characterised, the spatiotemporal expression patterns of genes governing sucrose transport and catabolism across distinct cell types remain poorly understood. Here, we present a high‐resolution spatiotemporal transcriptomic atlas of early soybean seed development, spanning the globular, heart, and cotyledon stages. We delineated 17 distinct cell types and identified 249 cell‐type marker genes. We further characterised the phloem region of the seed coat, a key compartment associated with sucrose transport and metabolism. Systematic profiling of sugar‐related genes revealed cluster‐specific and developmental stage‐dependent transcriptional patterns. We functionally characterised GmCWINV4, a phloem‐specific cell wall invertase that hydrolyses sucrose into glucose and fructose. Overexpression of *GmCWINV4* substantially increased seed size, weight, and oil content in soybean. We also identified two zinc finger homeodomain (ZF‐HD) transcription factors, GmZF‐HD27 and GmZF‐HD36, that directly activate *GmCWINV4* transcription by binding to ‘ATTA’ cis‐elements in its promoter. Moreover, we demonstrated that sucrose acts as an upstream signal that induces *GmZF‐HD36* transcription in a dose‐dependent manner. Collectively, these findings provide a comprehensive spatiotemporal transcriptomic resource for studying soybean seed development and reveal a previously unreported ZF‐HD–*GmCWINV4* regulatory module that modulates post‐phloem sucrose metabolism, thereby contributing to increased seed size and oil content. These findings offer mechanistic insights and potential targets for improving seed yield and nutritional quality in soybean.

## 1 Introduction

Soybean ( *Glycine max* [L.] Merr.) was first domesticated in China and has emerged as one of the most economically important crops worldwide (Graham and Vance 2003; Zhu et al. 2025). Soybean seeds are rich in vegetable oil (~20% of seed weight) and protein (~40%), making soybean a critical crop for food security and animal feed production (Graham and Vance 2003). Due to the economic importance and increased global demand for soybean products, boosting seed yield has become a key research focus in recent decades (Anderson et al. 2019). Advances in sequencing technologies and molecular biology have significantly accelerated progress in elucidating the molecular mechanisms governing seed development, seed size determination, and storage reserve accumulation (e.g., oil and protein) in soybean (Duan et al. 2023; Hu, Liu, Wei, et al. 2023). Consequently, several key genes regulating seed traits have been identified and functionally analysed in soybean, such as *Gibberellin 20‐oxidase* (*GmGA20OX*) (Lu et al. 2016), *GmSWEET10* (Wang et al. 2020), *Seed thickness 05* (*GmST05*) (Duan et al. 2022), *Protein, Oil, Weight, Regulator 1* (*POWR1*) (Goettel et al. 2022), *GmPLATZ* (Hu, Liu, Lu, et al. 2023), *Seed weight 16.1* (*GmSW16.1*) (Chen et al. 2023), *Seed width 17* (*GmSW17*) (Liang et al. 2024), *GmSW14* (Zhang, Li, et al. 2025), *Cell division cycle 7* (*GmCDC7*) (Yang et al. 2025), *GmSW19* (Zhang et al. 2026), and *GmMYB331* (Wang et al. 2026). However, most previous transcriptomic studies have relied on bulk RNA sequencing of whole seed tissues, which ignores the spatial complexity and cellular heterogeneity of developing soybean seeds. To further improve soybean seed yield and quality, it is imperative to gain a comprehensive understanding of the gene expression and regulatory networks occurring within seed subregions and cell types.

The soybean seed consists of three genetically distinct tissues: the maternally derived seed coat, the zygotic embryo, and the endosperm. Microscopy observations have revealed the principal developmental events and ultrastructural characteristics associated with early soybean seed development (Dute and Peterson 1992; Chamberlin et al. 1994; Miller et al. 1999). Like other major eudicot plants, soybean embryogenesis progresses through a series of successive stages: the zygote and proembryo stage, followed by the globular embryo stage, heart‐shaped embryo stage, cotyledon embryo stage, and mature embryo stage (Le et al. 2007). Concurrently, endosperm development typically proceeds through syncytial endosperm formation at the globular embryo stage, followed by cellularisation and differentiation at the heart‐shaped embryo stage, and cell death after the cotyledon embryo stage (Dute and Peterson 1992; Chamberlin et al. 1994). Alongside embryo and endosperm development, the seed coat differentiates into several subregions, that is, the inner integument, outer integument, endothelium, and epidermis, and accumulates pigments and mucilage at seed maturity (Miller et al. 1999). Therefore, early soybean seed development entails a sequence of distinct temporal and spatial stages, requiring precise coordination among various tissues and subregions. Recent advances in spatial transcriptomics and single‐cell sequencing have further enhanced our understanding of soybean seed development and functional compartmentalisation across distinct cell types (Pelletier et al. 2025; Zhang, Luo, et al. 2025). These studies have uncovered subregion‐specific or cell‐type‐specific marker genes and helped elucidate gene regulatory networks driving seed development, particularly within the endosperm and embryo (Pelletier et al. 2025; Zhang, Luo, et al. 2025).

Despite substantial progress in deciphering soybean seed development and seed‐trait control, high‐resolution spatial transcriptomic analysis of cell‐type‐specific genes involved in sugar metabolism and transport remains limited. This knowledge gap has hindered progress toward breeding for enhanced seed size and quality. Increasing evidence reveals that key developmental events in seeds, including the determination of seed size/weight and accumulation of storage reserves, are fueled by phloem‐imported sucrose (Weber et al. 1997; Pegler et al. 2023). In general, sucrose is initially unloaded into the phloem of the seed coat, followed by hydrolysis into hexose monomers catalysed by invertase (INV) or sucrose synthase (SUS). The resulting hexoses are subsequently transported into the filial tissues, namely the endosperm and embryo, through diverse sugar transporters, including members of the sucrose transporter (SUT or SUC), sugar transport protein (STP), and SWEET families (Li et al. 2017; Pegler et al. 2023). These sugar‐related genes are critical for soybean seed development, storage reserve accumulation, and seed yield. For example, the seed coat‐specific sucrose/hexose transporters GmSWEET10a and GmSWEET10b regulate seed size, oil content, and protein content (Wang et al. 2020). The Dt1 interacts with GmSWEET10a to negatively regulate the import of sucrose from the seed coat to the embryo, thereby modulating seed weight under long‐day photoperiods (Li et al. 2024). Two additional sugar transporters, *GmSWEET15a* and *GmSWEET15b*, are predominantly expressed in the endosperm, mediating sucrose export to the developing embryo; their knockout causes retarded embryo growth and severe seed abortion (Wang et al. 2019). Enhancing cell wall invertase (CWINV) activity by silencing the gene encoding its inhibitor Cell‐wall inhibitor of beta‐fructosidase (GmCIF1) substantially increased seed size and weight (Tang et al. 2017). Furthermore, two homologous cell wall invertases, GmCW11 and GmCW12, were recently shown to redundantly modulate seed yield and quality by regulating sugar metabolism (Mao et al. 2026). It is therefore important to characterise the spatiotemporal expression profiles of genes associated with sucrose metabolism and transport and to elucidate their roles in soybean seed development.

In this study, we constructed a high‐resolution spatiotemporal transcriptional atlas of early developing soybean seeds and systematically profiled the expression patterns of genes involved in sugar metabolism and transport. We functionally characterised the cell wall invertase gene *GmCWINV4*, which is specifically expressed in seed coat phloem and encodes an enzyme that catalyses the hydrolysis of sucrose into glucose and fructose. Overexpressing *GmCWINV4* produced significantly larger and heavier seeds with higher oil content in soybean. Notably, we identified two zinc finger homeodomain (ZF‐HD) transcription factors that bind to the *GmCWINV4* promoter and activate its expression. Our findings reveal a critical transcriptional regulatory module that governs carbon partitioning and seed size in soybean.

## 2 Results

### 2.1 A Spatiotemporal Transcriptomic Map Defines Distinct Subregions of Early Developing Soybean Seeds

Early seed development establishes the major seed structures and strongly influences final seed size (Li et al. 2019; Xu and Zhang 2023). To generate a comprehensive map of gene expression within distinct subregions of soybean seeds, we collected Williams 82 (Wm82) seeds at the globular, heart, and cotyledon stages of embryo development (designated as S1, S2, and S3, respectively; Figure 1a). We embedded the samples in OCT blocks for tissue sectioning, staining, and microscopy observation (Figure 1a). We identified the basic features of early developing soybean seeds, including the outer and inner seed coat, epidermis, hilum, embryo, and endosperm (see Figures 1b and S1). We then permeabilised the tissue sections and placed them onto a gene expression chip containing 2 million spots, each with a diameter of 2.5 μm. Transcripts were captured using poly(dT) oligonucleotides and sequenced on the BMKMANU S1000 platform (Figure 1a). The spatial positions of expressed genes were recorded using spatial barcodes, and genes expressed at each spot were distinguished by unique molecular identifiers (UMIs) (Figure 1a).

**FIGURE 1:**
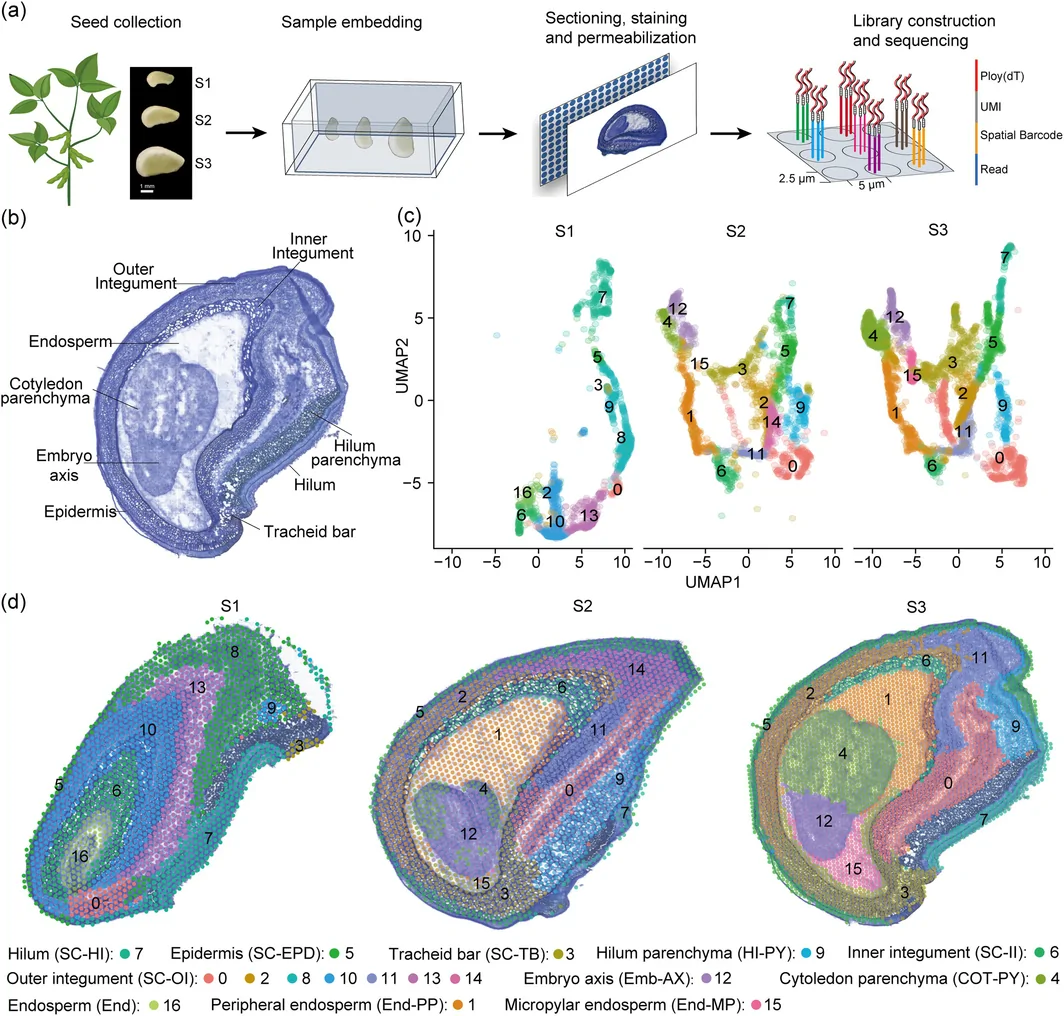
Spatial transcriptomics and clustering analysis in early developmental seeds of soybean. (a) Overview of sampling seeds stages and a workflow for spatial transcriptome sequencing (spRNA‐seq). S1, S2, and S3 denote the seeds sampled at the globular, heart, and cotyledon developmental stages, respectively. Scale bar: 1 mm. (b) Haematoxylin–eosin (HE) stained histological section of S3 stage seed. The known subregions of soybean seeds were delineated. (c) UMAP visualisation of the spatial transcriptomic clusters in S1 (left), S2 (middle), and S3 (right) soybean seeds. A total of 17 distinct clusters were identified across three stages. (d) Spatial mapping of cell clusters identified in panel (c) onto corresponding tissue sections (S1, S2, and S3). Each dot represents a spatial SupSpot, with colours indicating cells belonging to the same cluster. The corresponding anatomical regions for these clusters are indicated below.

After sequencing, we obtained 20.5 million reads (61.4 Gb), 88.2% of which uniquely aligned to the Wm82 genome. After spatial clustering at a resolution of 27 μm, we were able to accurately distinguish among different subregions of the soybean seed. A total of 8223 high‐quality SupSpots were obtained, with an average of 1160 genes detected per SupSpot; each SupSpot represents an aggregated analytical bin merged from raw spots (Figure S2a). We observed a strong positive correlation between gene numbers and UMIs (Pearson's correlation coefficient *r*_*p*_ > 0.98), along with a low percentage of chloroplast and mitochondrial genes (< 1%) per spot (Figure S2b). These results indicate that our spatial transcriptomic dataset from early‐stage soybean seeds is highly reliable and of high quality, making it suitable for analysis.

Upon clustering and dimensionality reduction using Seurat (see Methods), we identified 17 spatial clusters in total, with 10, 13, and 12 clusters in S1, S2, and S3, respectively (Figure 1c). Nine clusters were shared across all three stages of development. The S2 and S3 samples exhibited highly similar cell populations, except for Cluster 14, which was present only in S2, suggesting that these two stages of seed development share analogous developmental trajectories (Figure 1c). Based on the spatial coordinates of barcodes, we overlaid these clusters onto haematoxylin–eosin (HE)‐stained histological sections, allowing us to align the spatial clusters with known seed anatomical compartments (Figure 1b,d). As a result, we found that Clusters 4 and 12 correspond to cells from the embryonic cotyledon and axis, respectively; Clusters 1, 15, and 16 correspond to endosperm cells; Cluster 6 corresponds to inner integument cells; Cluster 3 corresponds to the tracheid bar; Clusters 5 and 7 correspond to epidermis and hilum cells; and the remaining clusters correspond to cells from the outer integument (Figure 1c,d). In summary, we constructed a spatiotemporal transcriptional map of early soybean seeds and successfully delineated their distinct subregions.

### 2.2 Spatial Transcriptomics Reveals Subregion‐Specific Biological Processes and Marker Genes

We detected 17 722 expressed genes in soybean seeds across the three developmental stages analysed here (Table S1). The number of expressed genes in different seed subregions ranged from 5525 (C3 at S1) to 15 386 (C8 at S1) (Figure S3). To explore the cellular processes that occur in each subregion, we identified cluster‐specific genes as those exhibiting a significantly higher proportion of expression within cells of that cluster compared to all other clusters. We identified between 45 (C2 at S2) and 1012 (C12 at S3) cluster‐specific genes (Figure S3 and Table S2). Functional gene ontology (GO) enrichment analysis revealed distinct biological processes that occur within specific subregions. These categories included ‘embryo morphogenesis’ and ‘pattern specification’ within the embryo proper (C4 and C12), ‘central cell differentiation’ and ‘cuticle development’ in the endosperm (C1), ‘suberin biosynthetic process’ in the inner integument (C6), ‘phytohormone catabolic process’ and ‘sucrose transport’ in the outer integument (C0 and C8), and ‘flavonoid biosynthetic process’ and ‘cuticular wax biosynthesis’ in the hilum and epidermis (C5 and C7) (Figure 2a).

**FIGURE 2:**
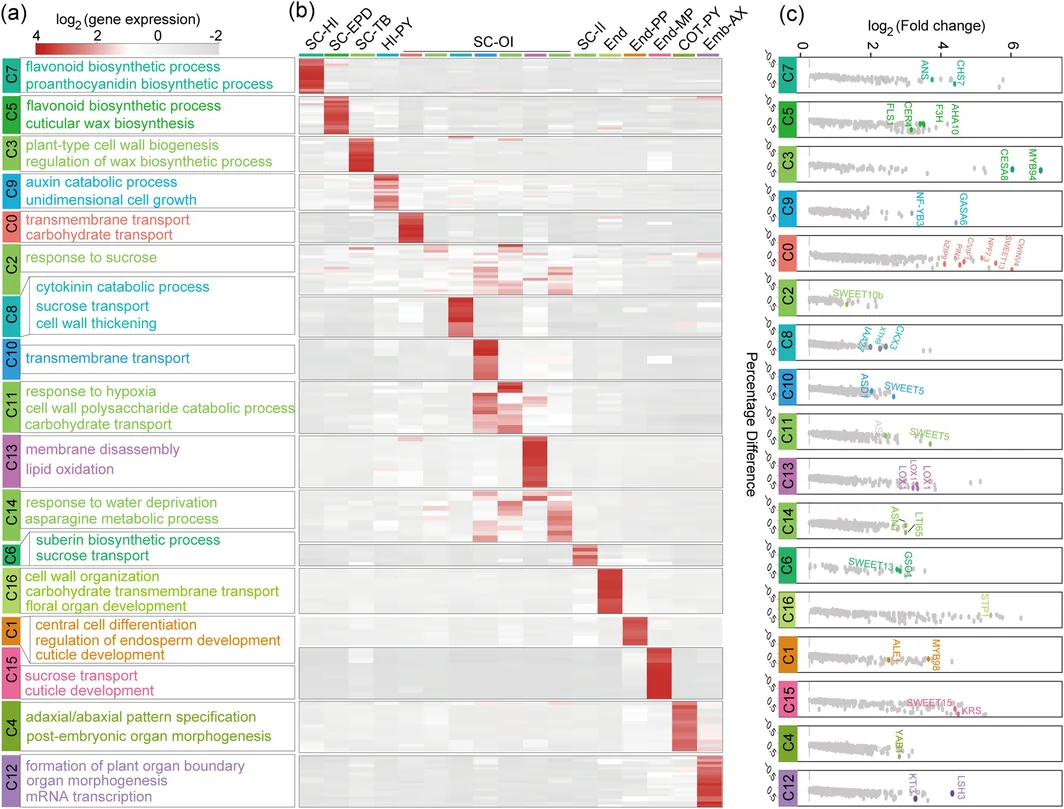
Functional characterisation of seed subregions and identification of marker genes. (a) Cluster‐specific GO terms based on functional enrichment analysis of cluster‐specific genes. (b) Cluster‐specific expression patterns of the marker genes (249 in total) for each cluster. SC‐HI: Seed coat hilum; SC‐EPD: Seed coat epidermis; SC‐TB: Seed coat tracheid bar; HI‐PY: Hilum parenchyma; SC‐OI: Seed coat outer integument; SC‐II: Seed coat inner integument; End: Endosperm; End‐PP: Peripheral endosperm; End‐MP: Micropylar endosperm; COT‐PY: Cytoledon parenchyma; Emb‐AXE: Embryo axis. (c) Visualisation of top cluster and known marker genes. Each row represents a distinct cell cluster (from C0 to C16). Grey points depict all detected cluster‐specific genes, while coloured points highlight the top marker genes for each cluster, labelled by gene name. The x‐axis shows log_2_‐transformed fold change in gene expression relative to other clusters.

We subsequently identified 249 marker genes, defined as the top five differentially expressed genes within each cluster (Table S3). These markers exhibited distinct cluster‐specific expression patterns, except for those from Clusters C2 and C14 (Figure 2b). Approximately 71.1% (177) of these marker genes were previously identified in the corresponding regions of soybean seeds (Zhang, Luo, et al. 2025; Pelletier et al. 2025) (Table S3). As shown in Figure 2c, the embryo marker gene *Kunitz Trypsin Inhibitor 3* (*GmKTi3*; Glyma.08G341500, Cluster C12) was previously characterised as embryo‐specific (Zhang, Luo, et al. 2025). The endosperm marker gene *GmSWEET15b* (Glyma.08G077200, Cluster C15), which is primarily expressed in the endosperm, encodes a transporter that mediates sucrose export from the endosperm to the developing embryo (Wang et al. 2019). Two other endosperm marker genes, *KERBEROS* (KRS; Glyma.02G292700, Cluster C15) and *ABNORMAL LEAF SHAPE1* (*ALE1*; Glyma.07G263500, Cluster C1), were expressed in the endosperm surrounding the embryo. Orthologous genes in Arabidopsis ( *Arabidopsis thaliana* ) are essential for embryo development (Tanaka et al. 2001; Moussu et al. 2017). The outer integument marker *GmSWEET10b* (Glyma.08G183500, Cluster C2) was previously shown to be specifically expressed in the seed coat (Wang et al. 2020). We also identified several marker genes, including the basic leucine zipper gene *bZIP9* (Glyma.14G139700) and *GmSWEET13* (Glyma.05G202700) within subregions C0 and C8 of the outer integument (Figure 2c and Table S3). Because these genes or their homologues are established phloem‐specific markers in soybean (Zhang, Luo, et al. 2025), Arabidopsis (Lee et al. 2025), and maize ( *Zea mays* ) (Bezrutczyk et al. 2018), their expression strongly supports the annotation of Clusters C0 and C8 as seed coat phloem cells. Notably, we also identified several previously unknown marker genes for each seed subregion with unknown functions (Table S3). These results indicate that our spatial transcriptomic data faithfully recapitulate the localisations of endogenous mRNAs in early developing soybean seeds.

### 2.3 Sugar Transport and Metabolism Genes Show Spatially and Temporally Distinct Expression During Early Seed Development

Seed development and growth are primarily driven by sucrose imported through the phloem (Weber et al. 1997; Pegler et al. 2023). Therefore, we investigated the spatiotemporal expression patterns of sugar‐associated genes across diverse cell types within soybean seeds. As shown in Figure 3a, sucrose is predominantly translocated from source to sink tissues via either apoplastic or symplastic pathways (Ruan 2014; Pegler et al. 2023). Within the apoplastic route, sucrose is hydrolysed by CWINV into glucose and fructose; these hexoses are subsequently imported into the cytosol via STPs or SWEETs (Figure 3a). Alternatively, sucrose can be directly transported into the cytosol via high‐affinity proton‐coupled sucrose transporters (SUTs or SUCs) or through plasmodesmata, where it may be further degraded by cytoplasmic invertase (CIN) or SUS (Figure 3a). Cytosolic sucrose can also be sequestered into vacuoles for hydrolysis by vacuolar invertase (VIN) (Ruan 2014).

**FIGURE 3:**
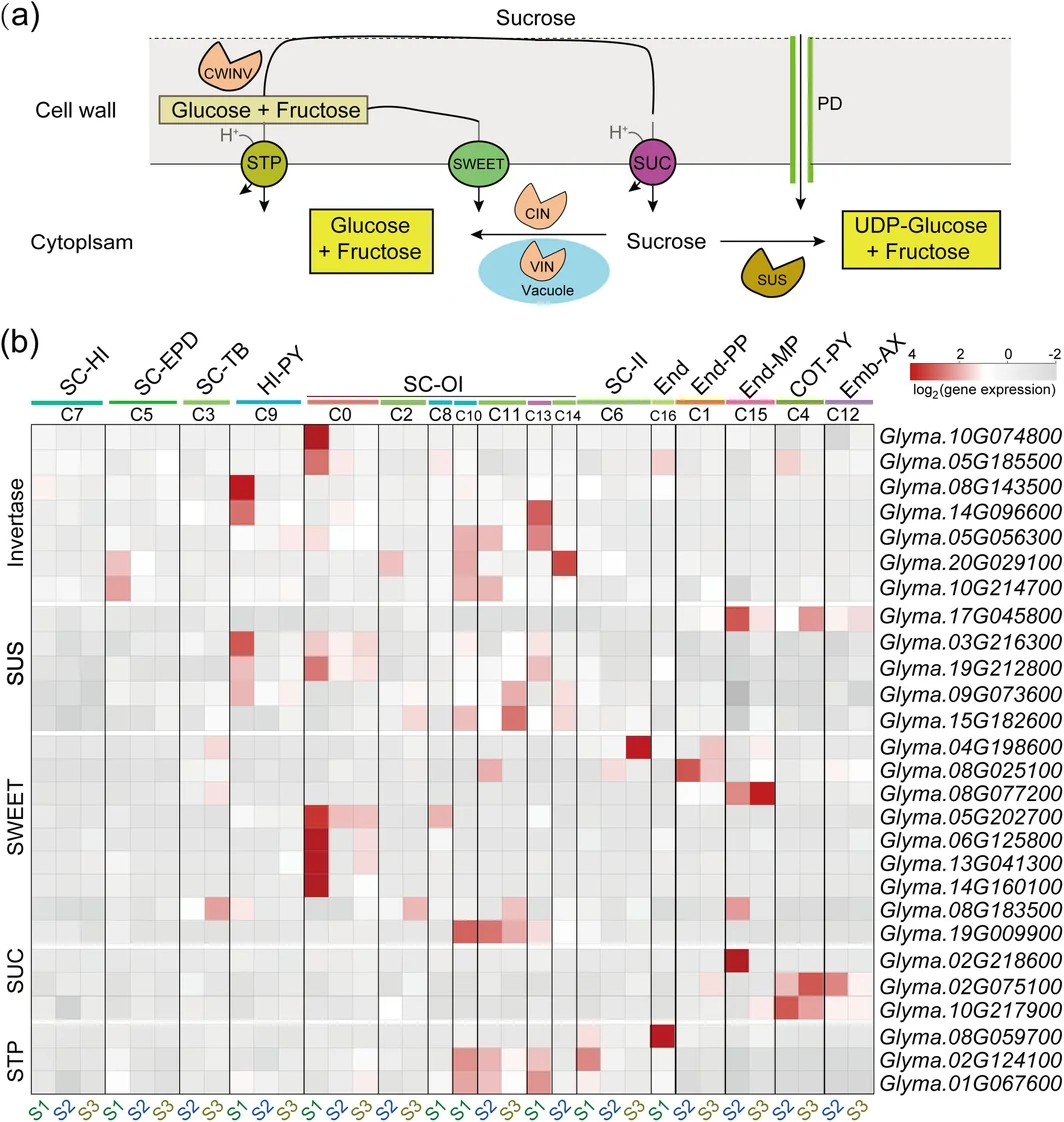
Identification of sugar‐related genes and their expression pattern within seed subregions across three developmental stages. (a) Diagram depicting the routes of sucrose unloading and metabolism in seed cells via apoplastic and symplastic pathways. Sucrose delivered by the phloem may be broken down by cell wall invertase (CWINV) into glucose and fructose, which subsequently enter the cytosol. Alternatively, sucrose is imported directly into the cytosol through SUC transporters or via plasmodesmata (PD). Inside the cytosol, sucrose can be cleaved by cytoplasmic invertase (CIN) or sucrose synthase (SUS), or it may be accumulated in vacuoles and later hydrolysed by vacuolar invertase (VIN). (b) Heatmap showing the expression pattern of sucrose transport‐ and catabolism‐genes within seed subregions across three developmental stages. SC‐HI: Seed coat hilum; SC‐EPD: Seed coat epidermis; SC‐TB: Seed coat tracheid bar; HI‐PY: Hilum parenchyma; SC‐OI: Seed coat outer integument; SC‐II: Seed coat inner integument; End: Endosperm; End‐PP: Peripheral endosperm; End‐MP: Micropylar endosperm; COT‐PY: Cytoledon parenchyma; Emb‐AXE: Embryo axis.

We identified 36 genes encoding invertases (16 CWINVs, 13 CINVs, and 7 VINVs), 12 genes encoding SUS, 47 genes encoding SWEETs, 15 genes encoding SUCs, and 32 genes encoding STPs across the genome (Table S4). Among these genes, seven *Invertase*, five *SUS*, nine *SWEET*, three *SUC*, and 3 *STP* genes exhibited subregion‐specific or stage‐specific expression patterns (Figure 3b and Table S5). Specifically, 10, 19, 4, 8, and 3 sugar‐related genes were highly expressed in the hilum and epidermis, outer integument, inner integument, endosperm, and embryo, respectively (Figure 3b and Table S5). For instance, *SWEET10b* (Glyma.08G183500) was specifically and highly expressed in the outer integument, while *SWEET15* (Glyma.08G077200) was exclusively expressed in the endosperm (Figure 3b). These expression patterns are consistent with previous reports (Wang et al. 2019, 2020). Notably, three *SUC* or *SUT* genes (Glyma.02G218600, Glyma.02G075100, and Glyma.10G217900) were specifically and highly expressed in the endosperm or embryo. Of these, *GmSUT1* (Glyma.10G217900) is specifically expressed in the embryo, and encodes a protein that regulates oil and protein accumulation in seeds (Sun et al. 2025). Furthermore, we identified several previously uncharacterised sugar metabolism‐ or sugar transport‐related genes with subregion‐specific expression patterns, including genes encoding endosperm‐specific sucrose transporters (STPs, Glyma.08G059700) and sucrose synthases (SUSs, e.g., Glyma.02G218600), as well as seed coat‐specific invertases (Glyma.14G096600, Glyma.08G143500, Glyma.05G185500, Glyma.10G074800) and SWEET transporters (Glyma.14G160100, Glyma.13G041300, Glyma.06G125800, Glyma.05G202700). Further investigation of these genes may uncover previously unknown roles in regulating soybean seed development and storage reserve accumulation.

### 2.4 *GmCWINV4* Encodes a Phloem‐Specific Cell Wall Invertase With Sucrose‐Hydrolysing Activity

We observed that 47.4% of sugar‐related genes (9/19) expressed in the outer integument were found in Cluster C0 (Figure 3b), indicating that active sugar metabolism and transport occur in this region. We identified Glyma.10G074800, a soybean homologue of Arabidopsis *CWINV4*, as a gene that is specifically expressed in Cluster C0 (Figure 4a) and the most prominent marker gene in this cluster (Figure 2c). Consistent with established cell types in soybean seeds (Zhang, Luo, et al. 2025, https://soybean‐atlas.com/), *GmCWINV4* was specifically expressed in the seed coat phloem (Figure S4a,b). RNA in situ hybridisation confirmed the region‐specific expression patterns of *GmCWINV4* (Figure 4b). *CWINV* genes are typically expressed in the phloem of sink tissues and play critical roles in determining sink strength and organ size (Ruan 2014; Pegler et al. 2023). Therefore, we selected *GmCWINV4* for investigation.

**FIGURE 4:**
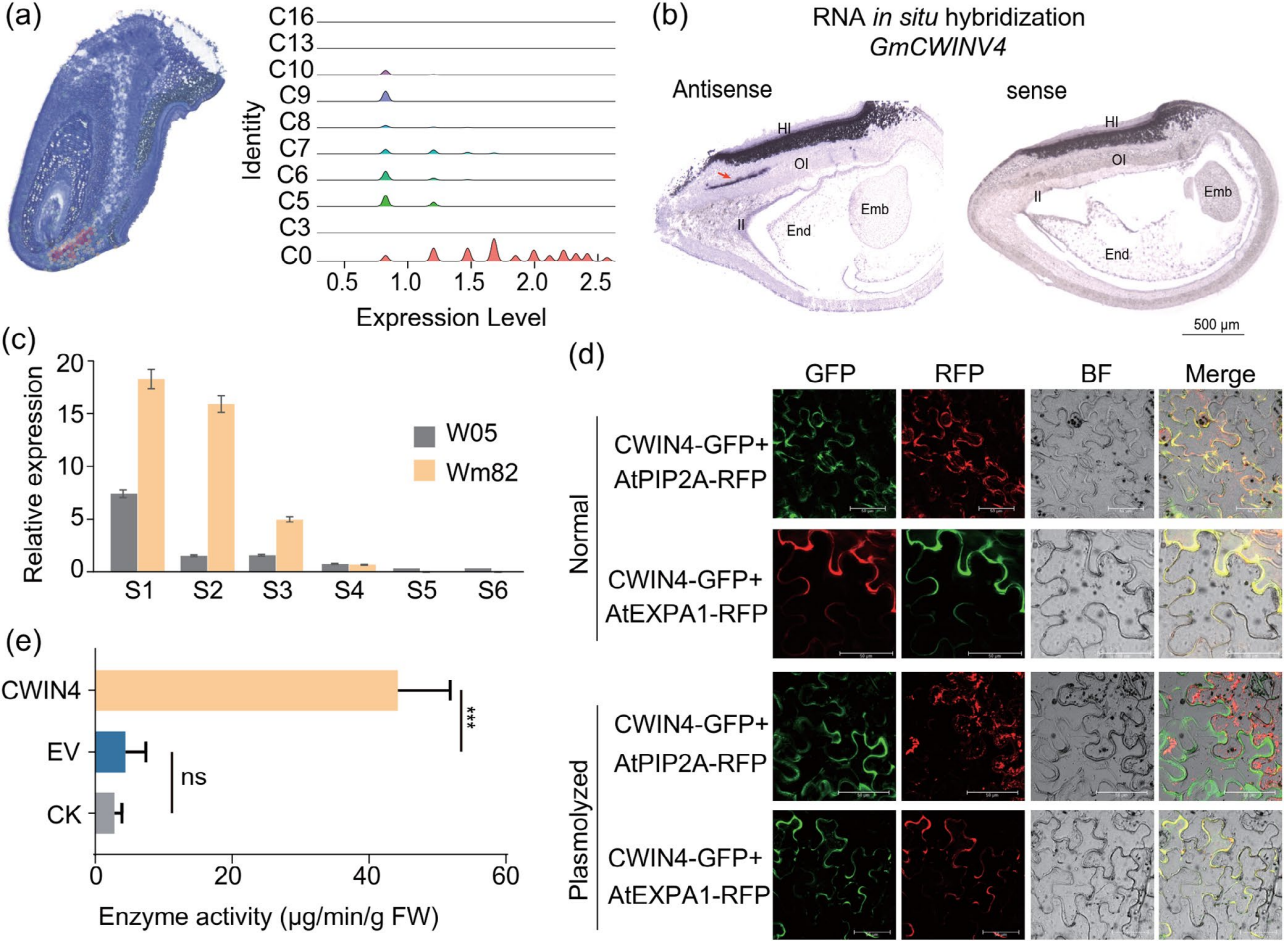
Expression pattern, subcellular localisation, and enzymatic activity of *GmCWINV4*. (a) Spatial Expression profile of *GmCWINV4* at S1 stage cell clusters, showing a specific and high expression level in Cluster C0. (b) RNA in situ hybridisation of *GmCWINV4* in developing soybean seed at S1 stage using antisense and sense probes. Red arrow denotes the expression region of GmCWINV4. HI: Hilum; OI: Outer integument; II: inner integument; End: endosperm; Emb: embryo. Scale bar: 500 μm. (c) The relative expression levels of *GmCWINV4* in seeds of cultivated soybean (Wm82) and wild soybean (W05) across developmental stages S1 to S6 (mean ± SD, *n* = 3). (d) Subcellular localisation of GmCWINV4 in tobacco leaves. Plasmolysis experiments verified that GmCWINV4 exhibits co‐localisation with the cell wall marker AtEXPA1‐RFP, while no overlap is observed with the plasma membrane marker AtPIP2A‐RFP. GFP: Green fluorescence protein; RFP: Red fluorescence protein; BF: Bright field. Scale bar: 50 μm. (e) Enzymatic activity assay of GmCWINV4. Transient overexpression of *GmCWINV4* in tobacco leaves significantly increases invertase activity compared to the empty vector (EV) and control (CK) (mean ± SD, *n* = 9. One‐way ANOVA followed by Tukey's HSD test for pairwise comparisons, ***p < 0.001; ns, not significant).

Reverse‐transcription quantitative RT‐PCR (RT‐qPCR) further validated the expression of *GmCWINV4* in early developing seeds from stages S1 to S3 (Figure 4c). Interestingly, this gene was expressed at significantly higher levels in the seeds of cultivated soybean (Wm82) than in those of wild soybean (W05) (Figure 4c). According to a public gene expression database (https://ngdc.cncb.ac.cn/soyomics), *GmCWINV4* is preferentially or exclusively expressed in reproductive organs such as flowers and seeds (Figure S4c). We investigated the subcellular localisation of GmCWINV4 via transient expression in *Nicotiana benthamiana* leaves. We first confirmed the localisation of two well‐characterised marker proteins: the plasma membrane marker PLASMA MEMBRANE INTRINSIC PROTEIN 2 (AtPIP2A) and the cell wall marker EXPANSIN 1 (AtEXPA1) (Figure S5). Transient co‐expression of *GmCWINV4‐GFP* with *AtPIP2A‐RFP* or *AtEXPA1‐RFP* in *N. benthamiana* leaves showed that GmCWINV4‐GFP appeared to co‐localise with both marker signals; after plasmolysis, GmCWINV4‐GFP overlapped with the cell wall marker AtEXPA1‐RFP but not with the plasma membrane marker AtPIP2A‐RFP, supporting cell wall localisation (Figure 4d).

To assess the hydrolytic activity of GmCWINV4 on sucrose, we conducted enzyme activity assays using the 3,5‐dinitrosalicylic acid (DNSA) method, in which DNSA reacts with reducing sugars to produce a brown‐red chromogenic amine derivative. We prepared crude protein extracts from the cell walls of *N. benthamiana* leaves expressing *GmCWINV4* and incubated them with sucrose as the substrate. We then quantified the resulting reducing sugars using the DNSA method. We observed a distinct brown‐red coloration in crude enzyme extracts derived from *N. benthamiana* leaves expressing *GmCWINV4* (Figure S6), suggesting that GmCWINV4 hydrolysed the supplied sucrose into reducing sugars. Furthermore, GmCWINV4 activity (~44.234 μg/min/g fresh weight [FW]) was significantly elevated in the cell walls of *N. benthamiana* leaves expressing *GmCWINV4* compared to non‐infiltrated leaves (~2.829 μg/min/g FW) and the empty vector control (~4.428 μg/min/g FW) (Figure 4e). Therefore, we identified and functionally analysed the phloem‐specific cell wall invertase GmCWINV4 in soybean seeds.

### 2.5 GmCWINV4 Simultaneously Enhances Seed Size and Oil Content

To investigate the roles of GmCWINV4 in soybean seeds, we obtained two independent soybean overexpression lines, *GmCWINV4‐*OE2 and *GmCWINV4‐*OE5, by introducing overexpression vectors driven by the soybean *Ubiquitin* promoter (Figure S7). The seeds of the overexpression lines were significantly larger than those of Wm82 (Figure 5a). Specifically, seed length, width, and thickness were 6.0%, 8.0%, and 11.6% greater, respectively, in the *GmCWINV4*‐overexpression lines than in Wm82 (Figure 5b–d), and hundred‐seed weight was 24.2% greater (Figure 5e). Cytological observations showed that cotyledon cells were substantially larger in the *GmCWINV4*‐overexpression lines than in Wm82 (Figures 5f,g and S8), suggesting that the greater seed size may result from expansion of the cell area. Analysis of seed constituents revealed altered seed composition in the *GmCWINV4*‐overexpression lines: seed oil content was 13.3% higher than in Wm82 (Figure 5h), whereas protein content was 4.8% lower (Figure 5i). Moreover, the overexpression lines contained elevated sucrose, glucose, and fructose levels in mature seeds (Figure 5j–l). These results demonstrate that overexpressing *GmCWINV4* leads to larger and heavier seeds with higher oil and soluble sugar contents but lower protein content.

**FIGURE 5:**
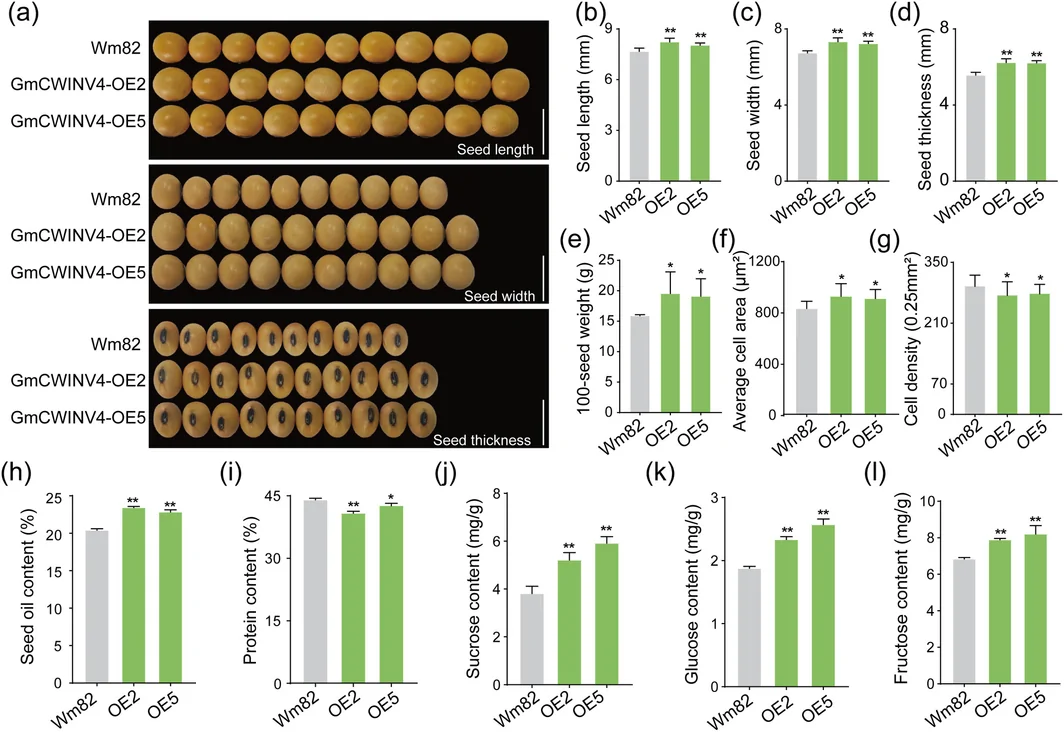
Overexpression of *GmCWINV4* enhances the seed size and oil content. (a) Morphological characteristics of mature seeds from wild‐type (Wm82) and *GmCWINV4* overexpression lines (OE2 and OE5), with representative seeds arranged to illustrate variations in length, width, and thickness. Scale bar: 1 cm. (b–d) Quantitative analysis of seed length (b), width (c), and thickness (d) (*n* = 20). (e) Quantification of hundred‐seed weight in Wm82 and *GmCWINV4*‐overexpressing lines (*n* = 5). (f, g) Histological analysis of seed cotyledons, showing cell density (f) and average cell area (g). (h, i) Quantification of seed oil content (h) and protein content (i) in mature seeds (*n* = 8). (j–l) Quantification of soluble sugar contents, including sucrose (j), glucose (k), and fructose (l) in mature seeds (*n* = 3). Data are shown as means ± standard errors. Asterisks denote statistically significant differences between overexpressing lines and wild‐type (Wm82) plants (*p < 0.05, **p < 0.01; Student's *t*‐test).

To characterise haplotype variation of *GmCWINV4*, we extracted genetic variants within its 2‐kb promoter and genic regions from SoyMD population resequencing data (https://yanglab.hzau.edu.cn/SoyMD/#/variation). After filtering out variants with a minor‐allele frequency (MAF) < 0.05, 11 haplotypes were identified (Figure S9a). Median‐joining network clustered these haplotypes into four major clades (H_I‐H_IV; Figure S9b). We found that H_IV was predominant in wild soybean (49%), whereas H_I dominated in landraces (83%) and improved cultivars (94%) (Figure S9c). Such marked haplotype‐frequency shifts between wild and cultivated populations show that *GmCWINV4* could be a candidate locus underlying selection during domestication. Phenotypic association analysis revealed significant differences in seed oil and protein contents across the four major haplotype groups (Figure S9d). Accessions carrying H_I exhibited the highest seed oil content, whereas H_IV accessions accumulated the lowest oil levels. By contrast, H_I‐carrying accessions displayed relatively low seed protein content, and H_IV accessions showed significantly elevated protein concentration (Figure S9d). These results point to the H_I alleles of *GmCWINV4* as promising targets for improving seed oil content in modern soybean cultivars.

### 2.6 GmZF‐HD27 and GmZF‐HD36 Transcriptionally Activates *GmCWINV4* Expression

To explore the transcriptional regulatory mechanisms underlying phloem‐specific *GmCWINV4* expression, we looked for proteins that bind to its promoter (a 922‐bp fragment) by performing a yeast one‐hybrid (Y1H) screening. This analysis yielded 77 positive clones (Table S6). After Sanger sequencing and BLAST analysis, we identified two candidate Zinc Finger Homeodomain (ZF‐HD) transcription factors that might bind to the *GmCWINV4* promoter (Table S6). These were designated GmZF‐HD27 (Glyma.09G238400) and GmZF‐HD36 (Glyma.14G179600), following a previous report (Rizwan et al. 2025). Both genes exhibited higher expression in Clusters C0 and C8 (seed coat phloem cells) relative to other subregions at stage S1 (Figure S10). Subcellular localisation assays in *N. benthamiana* leaf epidermal cells showed that RFP‐tagged GmZF‐HD27 and GmZF‐HD36 colocalised with DAPI‐stained nuclei, confirming their nuclear localisation (Figure 6a).

**FIGURE 6:**
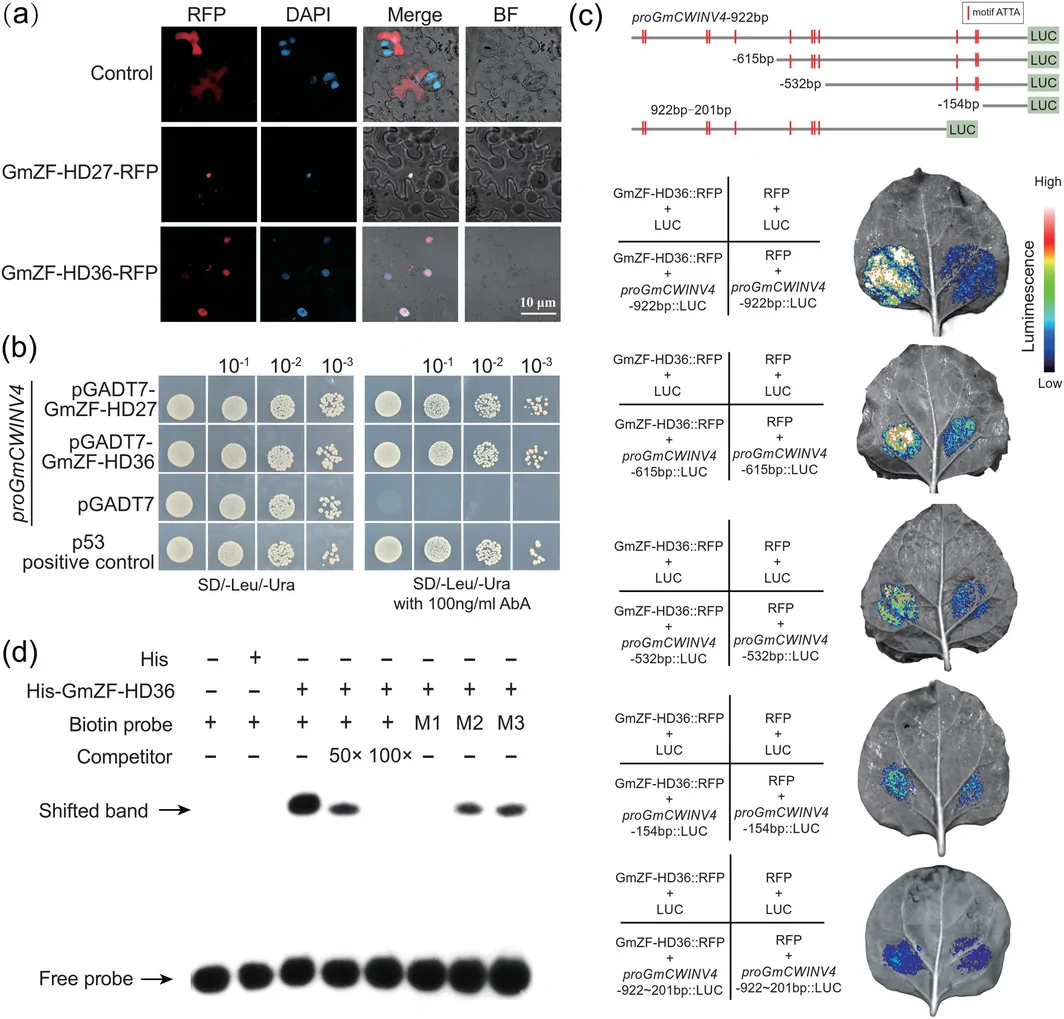
Transcriptional regulation of *GmCWINV4*. (a) Subcellular localisation of GmZF‐HD27 and GmZF‐HD36 in tobacco epidermal cells. DAPI (4′,6‐diamidino‐2‐phenylindole) staining indicates the cell nucleus. RFP: Red fluorescence protein; BF: Bright field. Scale bar: 10 μm. (b) The yeast one‐hybrid (Y1H) assays indicate the binding between GmZF‐HD27/36 transcription factors and the *GmCWINV4* promoter. (c) Transcriptional activation of GmZF‐HD36 on a series of 5′‐terminal sequentially truncated promoters of *GmCWINV*4 (−922 bp, −615 bp, −532 bp, and −154 bp), together with an internal deletion construct (−922 bp to −201 bp), according to the positional distribution of the ‘ATTA’ cis‐regulatory motif for dual‐luciferase reporter assays. (d) Electrophoretic mobility shift assay (EMSA) of his‐GmZF‐HD36 fused protein binding to biotin probes with corresponding motifs and mutant sequences (M1, M2, M3). − the absence of corresponding probes; + the presence of corresponding probes; competitor: Corresponding motif sequences without biotin.

To validate the interaction between GmZF‐HD27, GmZF‐HD36, and the *GmCWINV4* promoter, we conducted a targeted Y1H assay. Accordingly, we cloned the *GmCWINV4* promoter into the bait vector pAbAi and inserted the full‐length coding sequences of *GmZF‐HD27* and *GmZF‐HD36* individually into the prey vector pGADT7. The empty vector pGADT7 served as a negative control, and pGADT7‐p53 served as a positive control. As shown in Figure 6b, yeast cells transformed with the negative control showed no colony growth on selection medium containing 100 ng/mL Aureobasidin A (AbA). Conversely, the positive control exhibited vigorous colony formation, validating the robustness of the Y1H assay system. Transformed cells harbouring the *GmCWINV4* bait and the pGADT7‐GmZF‐HD27 or pGADT7‐GmZF‐HD36 construct exhibited colony formation on selective medium, indicating that GmZF‐HD27 and GmZF‐HD36 directly bind to the *GmCWINV4* promoter (Figure 6b).

ZF‐HD transcription factors show a binding preference for the cis‐regulatory element 5′‐ATTA‐3′ in soybean (Park et al. 2010). Our analysis identified 12 such ‘ATTA’ motifs within the 922‐bp promoter region of *GmCWINV4* (Figure 6c). To identify the critical binding sites, we generated a series of 5′ truncations of the *GmCWINV4* promoter, with fragment lengths designed based on the positional distribution of the ‘ATTA’ motifs (Figure 6c). In dual‐luciferase reporter assays, both GmZF‐HD36 and GmZF‐HD27 transactivated the *GmCWINV4* promoter following the deletion of ‘ATTA’ motifs spanning positions −532 bp to −922 bp relative to the transcription start site (Figures 6c, S11 and S12a,b). Conversely, the most proximal 154 bp promoter fragment exhibited no significant LUC activity (Figures 6c, S11 and S12a,b). These observations suggest that GmZF‐HD36 and GmZF‐HD27 predominantly bind to the ‘ATTA’ motifs located within the −532 bp to −154 bp region of the *GmCWINV4* promoter, which harbours three ‘ATTA’ motifs spanning −198 bp to −154 bp. Furthermore, when this region (−198 bp to −154 bp) containing the three ‘ATTA’ motifs was deleted from the *GmCWINV4* promoter, we failed to detect LUC activation (Figures 6c, S11 and S12a,b), thereby delineating the core regulatory sequence.

We performed an electrophoretic mobility shift assay (EMSA) to further validate the binding of GmZF‐HD36 and GmZF‐HD27 to the GmCWINV4 promoter region spanning −198 bp to −154 bp. Recombinant purified His‐GmZF‐HD36/27 bound to the labelled probe, while this binding was completely eliminated by the addition of a 100‐fold molar excess of unlabeled competitor probes (Figures 6d and S12c). To precisely identify the core binding sites, we designed three mutated probes (M1, M2, and M3) targeting the ‘ATTA’ motifs (Table S7). M1 contained mutations in all three ‘ATTA’ sequences, M2 contained a mutation in a single ‘ATTA’ site, and M3 contained mutations in two adjacent ‘ATTA’ sites. These probes were further examined via EMSA using recombinant His‐GmZF‐HD36 protein. We found that His‐GmZF‐HD36 did not bind to the M1 probe, whereas its binding to the M2 and M3 probes was weaker than that to the intact sequence (Figure 6d), suggesting that all three ‘ATTA’ sites are required for efficient activation of *GmCWINV4* expression. Similarly, His‐GmZF‐HD27 failed to bind to the M1 probe carrying mutations in all three ‘ATTA’ sequences (Figure S12c). Together, these results reveal that GmZF‐HD27 and GmZF‐HD36 directly bind to the *GmCWINV4* promoter to activate its transcription.

### 2.7 Sucrose Induces *GmZF‐HD36* Expression in Soybean Seeds

Considering that GmZF‐HD27 and GmZF‐HD36 activate the expression of *GmCWINV4* in phloem parenchyma cells of the soybean seed outer integument, the site of sucrose unloading, we reasoned that sucrose might induce *GmZF‐HD27* and *GmZF‐HD36* expression to regulate sucrose hydrolysis. To test this hypothesis, we isolated developing seeds at the S1 stage, cultured them in liquid Murashige and Skoog (MS) medium in vitro, and supplied them with sucrose (1% or 3%, w/v) for 8 h. RT‐qPCR analysis showed that sucrose treatment failed to trigger transcriptional induction of *GmZF‐HD27* but markedly upregulated *GmZF‐HD36*, relative to the no‐sucrose control (Figure S13a).

Finally, to further corroborate the sucrose‐induced transcriptional activation of *GmZF‐HD36*, we performed dual‐luciferase reporter assays in *N. benthamiana* leaves using a reporter construct *proGmZF‐HD36*::LUC, in which the native *GmZF‐HD36* promoter was fused upstream of the *LUC* reporter gene (Figure S13b–e). To rule out the effects of osmotic stress, we included parallel treatments with 1% or 3% mannitol (w/v) (Figure S13c). Both 1% and 3% sucrose treatments generated distinct LUC signals, with maximal promoter activity observed under 3% sucrose treatment (Figure S13d,e), whereas there was no detectable induction under mannitol treatment or in the negative control (Figure S13b–e), confirming the specificity of sucrose‐mediated *GmZF‐HD36* promoter induction. Collectively, these results suggest that sucrose acts as an upstream signal inducing transcription of *GmZF‐HD36*.

## 3 Discussion

Spatially compartmentalised sucrose transport and metabolism within discrete seed domains profoundly affect the developmental progression of soybean seeds. This tightly orchestrated sugar flux shapes final seed biomass and the accumulation of storage compounds (Pegler et al. 2023). In this study, we generated a high‐resolution spatial transcriptome to systematically profile gene expression, especially sugar‐related gene expression, during early soybean seed development. We delineated 17 seed cell types (Figure 1), most of which recapitulate previously reported seed cell populations and their biological functions, especially clusters derived from the embryo and endosperm (Zhang, Luo, et al. 2025; Pelletier et al. 2025). Despite this overall consistency with prior reports, our spatiotemporal transcriptomics uncovered the spatial complexity and functional diversity within the outer integument (Figure 1). Clusters residing in the outer integument exhibited divergent functional specialisations, including flavonoid and cuticular wax biosynthesis, carbohydrate and sucrose transport, hormone metabolism, and cell wall biogenesis (Figure 2a). Focusing on sugar metabolism and transport during seed development, we identified a suite of sugar‐related genes, including *INVs*, *SUSs*, *SWEETs*, *SUCs*, and *STPs*, that are predominantly expressed in the outer integument and exhibit cell‐type‐specific spatiotemporal expression patterns (Figure 3b). For example, *SWEET10b*, a marker gene of Cluster C2 representing thick‐walled parenchyma cells of the outer integument (Pegler et al. 2023), encodes a transporter for sucrose and hexoses that facilitates sugar allocation from the seed coat to the embryo (Wang et al. 2020). GO enrichment analysis of Cluster C2 also revealed enrichment for terms associated with sucrose response (Figure 2a). Another marker gene, *SWEET13*, detected in outer‐integument Clusters C0 and C8, exhibits conserved phloem‐specific expression across plant species and acts as a key mediator of apoplasmic phloem loading (Bezrutczyk et al. 2018; Chen et al. 2022; Lee et al. 2025; Pelletier et al. 2025). Consistently, genes within C0 and C8 are mainly associated with carbohydrate and sucrose transport (Figure 2a), and nearly half of outer‐integument sugar‐related genes are highly enriched specifically in this C0 phloem domain (Figure 2b). This finding aligns with early physiological evidence demonstrating that seed coat phloem serves as a critical hub for sucrose unloading and translocation in developing soybean seeds (Gifford and Thorne 1986). Notably, many clusters identified in the outer integument (e.g., C9–C14) remain functionally unannotated, and their cellular identities are still poorly characterised. Collectively, this spatial transcriptomic resource lays a foundation for defining distinct seed cell types and uncovering the genetic basis governing seed development and nutrient translocation within seed‐coat subregions.

Within the outer‐integument seed phloem, we identified the gene encoding cell wall invertase GmCWINV4, which represents the most prominent marker gene of seed phloem (see Figure 4b). Subcellular localisation and enzyme activity assays revealed that GmCWINV4 functions as a cell wall invertase that hydrolyses sucrose (see Figure 4d,e). Accumulating evidence indicates that CWINV‐mediated sucrose hydrolysis is indispensable for post‐phloem assimilate utilisation in sink organs and is tightly associated with reproductive success, organ growth, and crop yield performance (Ruan 2014; Pegler et al. 2023). For instance, the loss of function of the *Miniature 1* gene, which encodes a cell wall invertase in maize seeds, resulted in an approximately 70% drop in seed weight (Kang et al. 2009). The rice ( *Oryza sativa* ) gene *GRAIN INCOMPLETE FILLING 1* (*GIF1*), encoding a cell wall invertase, was isolated from early developing grains; its overexpression driven by its native promoter led to greater grain production (Wang et al. 2008). The constitutive expression of genes encoding cell wall invertases from Arabidopsis, rice, or maize significantly enhanced both grain yield and starch content in transgenic maize plants (Li et al. 2013). Conversely, the specific genetic repression of *CWIN2* and *CWIN4* in Arabidopsis leads to ovule and seed abortion (Liao et al. 2020).

To investigate the function of *GmCWINV4* in soybean seeds, we overexpressed this gene in Wm82. Overexpressing *GmCWINV4* resulted in significantly larger and heavier seeds that accumulated more oil and soluble sugars (including sucrose, glucose, and fructose) but significantly less protein (Figure 5). Consistent with this result, a recent study revealed that the homologous genes *GmCWI11* and *GmCWI12* also strongly affect soybean seed size and sugar content (Mao et al. 2026). Cytological observations showed that greater seed size may result from expansion of the cell area (Figure S8). Elevated invertase‐dependent sucrose hydrolysis generates hexoses that may drive cell expansion through osmotic adjustment and carbon provision for cell‐wall biosynthesis (Ruan et al. 2010; Wang and Ruan 2013). Apart from metabolic supply, hexoses originating from GmCWINV4 activity could also act as signal molecules to modulate cell expansion, potentially crosstalking with auxin‐dependent regulatory pathways to coordinate cell enlargement (Wang and Ruan 2013). In addition, we observed that numerous genes encoding SWEETs were coordinately expressed with *GmCWINV4* (Figure 3b), suggesting that this synergistic relationship supplies hexoses to sink tissues. Similarly, the coordinated expression of *CWINV* and additional sugar transporter genes has been observed in diverse plant species, where it plays a critical role in regulating early seed development (Pegler et al. 2023). Collectively, these findings reveal a critical role for GmCWINV‐mediated sucrose metabolism in determining seed size and quality.

Identifying cis‐motifs and regulators of *CWINV* transcription will improve our understanding of carbon partitioning in developing seeds. To date, the transcriptional regulation of *CWINV* genes has been characterised in diverse plant species (Li et al. 2017). For example, two *CWINV* genes in tomato ( *Solanum lycopersicum* ; *Lycopersicon Invertase 5* [*Lin5*] and *Lin7*) are specifically expressed in reproductive organs, and their promoters harbour multiple cis‐acting elements that are responsive to plant hormonal treatments (Proels et al. 2003). Another tomato *CWINV* gene (*Lin6*) is specifically expressed in the phloem, and its expression is induced by sucrose (Proels and Roitsch 2009). In Arabidopsis, five transcription factors were shown to regulate the expression of *CWINV2* and *CWINV4*: AUXIN RESPONSE FACTOR 6 (ARF6), ARF8, MYB21, APETALA 3 (AP3), and CRABS CLAW (CRC) (Li et al. 2021). Additionally, the heterologous expression of soybean *bZIP123* increased lipid contents in the seeds of transgenic Arabidopsis plants by directly regulating the expression of sugar‐related genes including *CWINV1*, *CWINV3*, and *CWINV6* (Song et al. 2013).

In this study, Y1H screening identified GmZF‐HD36 and GmZF‐HD27 as upstream regulators of the phloem‐specific gene *GmCWINV4*. Both transcription factor genes were highly expressed in the seed phloem compared to other subregions (Figure S10). Furthermore, dual‐luciferase reporter assays and EMSAs demonstrated that GmZF‐HD36 and GmZF‐HD27 directly activate the transcription of *GmCWINV4* by binding to the ‘ATTA’ cis‐motifs within its promoter (Figures 6 and S12). Interestingly, we observed that sucrose induced the expression of *GmZF‐HD36*, suggesting its promoter likely contains motifs responsive to sucrose or sugar signalling pathways. These results indicate partial functional redundancy between GmZF‐HD36 and GmZF‐HD27 in activating *GmCWINV4* expression, alongside distinct responsiveness to upstream sugar signalling. It appears that GmZF‐HD36 acts as the primary effector linking sucrose signals to *GmCWINV4* expression, while GmZF‐HD27 participates in this regulatory circuit through sucrose‐independent pathways. The roles of GmZF‐HD27 and GmZF‐HD36 in regulating soybean seed development and related traits merit further investigation. Additionally, CWINV activity might be regulated at the post‐translational level through a protein–protein interaction with an inhibitory protein, that is, a cell wall invertase inhibitor like CIF (Li et al. 2021). Indeed, silencing *GmCIF1* expression significantly elevated CWINV activity and improved seed weight in soybean (Tang et al. 2017). In this study, we identified a marker gene encoding CIF in the seed phloem (Figure 2c), implying that GmCWINV4 is subject to precise regulation of sucrose metabolism and sink strength during soybean seed development.

In conclusion, we propose a working model describing the phloem‐specific function of GmCWINV4 and its regulatory mechanism (Figure 7). During the early phases of seed development, sucrose is translocated to and unloaded in the phloem, thereby stimulating the expression of *GmZF‐HD* genes via an unknown mechanism that likely involves sugar signalling pathways. Consequently, GmZF‐HD27 and GmZF‐HD36 specifically bind to the ‘ATTA’ motifs in the *GmCWINV4* promoter, activating its expression. The high levels of GmCWINV4 hydrolyse sucrose into glucose and fructose, promoting seed enlargement and oil deposition in soybean seeds (Figure 7). While our work establishes the regulatory framework, several key genetic validations remain to be investigated in future work. Specifically, the absence of *GmCWINV4* knockout lines limits our ability to fully validate its endogenous physiological function. In addition, the regulatory roles of GmZF‐HD27 and GmZF‐HD36 are currently supported only by molecular interaction assays, without *in planta* genetic evidence in soybean. Future characterisation of CRISPR‐Cas9 knockout mutants will strengthen this regulatory model and clarify the genetic functions of these genes during seed development.

**FIGURE 7:**
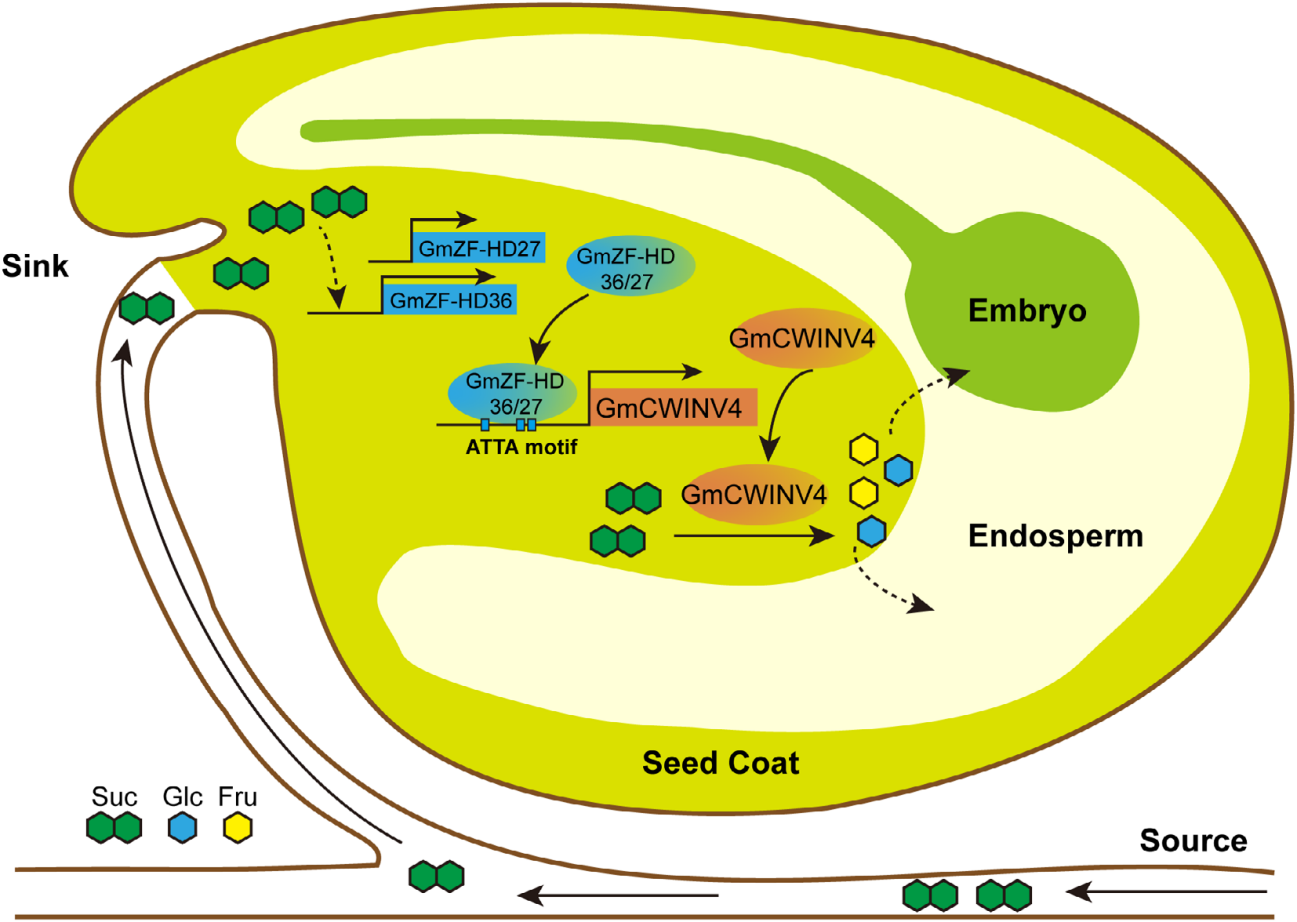
Proposed working model illustrating a GmZF‐HD‐*GmCWINV4* regulatory module governing carbon partitioning and seed size in developing soybean seeds. Sucrose (Suc) synthesised in photosynthetic source tissues is translocated into the seed coat phloem region of developing soybean seeds. Sucrose accumulation induces the expression of *GmZF‐HD36* transcription factor, which directly binds to conserved ‘ATTA’ cis‐elements within the *GmCWINV4* promoter to transcriptionally activate *GmCWINV4*. Meanwhile, another TF, GmZF‐HD27, which is not induced by sucrose, also binds to the ‘ATTA’ cis‐elements and activates *GmCWINV4* expression. Elevated cell wall invertase activity hydrolyses phloem‐imported sucrose into glucose (Glc) and fructose (Fru), which further modulates seed size and lipid accumulation.

## 4 Materials and Methods

### 4.1 Plant Growth Conditions and Sample Collection

Soybean cultivar Williams 82 was grown in the greenhouse under a 16‐h day (28°C)/8‐h night (22°C) condition at Kunming Botanical Garden (Kunming Institute of Botany, Kunming, Yunnan, China). Seeds were harvested at the globular (S1), heart (S2), and cotyledon (S3) developmental stages and carefully placed on a unified OCT‐embedding block (cold optimum cutting temperature, SAKURA, Cat#:4583), which was snap‐frozen in isopentane that was pre‐chilled with liquid nitrogen. Samples were then immediately overlaid with OCT and stored at −80°C for subsequent cryo‐sectioning.

### 4.2 Spatial Transcriptome Experiments and Sequencing

Briefly, the sample embedded in OCT was sectioned at 10 μm by a freezing microtome and adhered to the surface of a chilled BMKMANU S1000 Tissue Optimisation Slide. Permeabilisation was performed using the commercial permeabilisation enzyme mix from the BMKMANU Tissue Optimisation Reagent kits (Biomarker Technologies Corporation, Beijing, China) according to the manufacturer's instructions (http://www.bmkmanu.com/download). Thereafter, the sample was sectioned again from same OTC‐embedding block and placed on Gene Expression Slides (6.8 mm × 6.8 mm) containing 2 000 000 spots each of 2.5 μm diameter. The expression slides were processed according under the optimal conditions. Subsequently, mRNAs were released from the tissue slice and captured by designed barcode sequences that includes a spatial barcode, unique molecular identifiers (UMI) and Poly(dT)VN (for 3′ polyA mRNA enrichment). In each spot, the first‐ and second‐strand cDNAs were generated using BMKMANU S1000 Gene Expression kits, and the cDNA library was constructed using BMKMANU S1000 Library Construction Kit. The concentration and quality of the cDNA library were assessed on Qubit (Invitrogen) and High Sensitivity Agilent 2100 Bioanalyser (Agilent Technologies), respectively. Finally, cDNA library was sequenced on the Illumina NovaSeq 6000 with 150‐bp paired‐end reads (Biomarker Technologies Corporation, Beijing, China). Besides, bright‐field images of tissue sections were taken at 20× magnification using Metafer Slide Scanning platform (MetaSystems) for subsequent spot visualisation and image alignment.

### 4.3 Spatial Transcriptomics Data Analysis

Raw reads were trimmed and quality filtered and trimmed by Trimmomatic (version 0.36) and evaluated with fastqc (version 0.11.9, https://www.bioinformatics.babraham.ac.uk/projects/fastqc). Clean data and bright field microscope section images were processed with BSTMatrix (version 2.2, http://www.biomarker.com.cn/zhizao/tools) software, which detected the captured tissue area on the chip and distinguished reads from each spot by spatial barcode information. Meanwhile, the clean data were mapped to the soybean Williams 82 genome (Wm82.a4.v1, https://phytozome.jgi.doe.gov) with STAR software (Dobin et al. 2013). Finally, UMI counts for each spot were used to remove PCR duplicates, and a gene‐spot expression matrix with multi‐level resolution was obtained by using BSTMatrix software with default parameters. Based on the bright‐field image of samples and spatial expression matrix, we obtained separately the Level 4 (27 μm resolution) matrix for soybean seed in globular, heart, and cotyledon stage using BSTViewer software (version 3.0, http://www.biomarker.com.cn/zhizao/tools).

### 4.4 Cell Clustering and Marker Genes Identification

We first filtered spots with fewer than 200 genes with the parameters: *min.features = 200* and converted the spatial expression matrix into a format suitable for Seurat‐compatible format using the CreateBmkObject.R script (https://www.bmkgene.com/software‐downloads/). Next, the converted object for each sample was subjected to the Seurat package (version 4.1.0; Stuart et al. 2019) for normalisation, dimensionality reduction, clustering and identification of differential genes. Briefly, we used negative binomial regression (SCTransform) to normalise UMI counts and screen highly variable genes with the parameter: *variable.features.n = 3000*. Principal component analysis (PCA) linear dimensionality reduction was then performed using RunPCA, and the resulting output was used for FindNeighbors (*dims = 1:30*) and FindClusters (resolution = 0.72) in Seurat to identify cell clusters. We finally visualised the clustering results using the UMAP algorithm (RunUMAP in Seurat).

Furthermore, we used AverageExpression method in Seurat to calculate the average expression level of genes in each cluster, and employed FindAllMarkers to identify the differentially expressed genes (DEGs) with the threshold of log_2_Fold_Change > 1.5 and *Q* value < 0.05. These DEGs were further subjected to gene ontology (GO) enrichment analyses using soybeanGDB website (https://venyao.xyz/soybeangdb/).

### 4.5 Subcellular Localisation and Enzyme Activity Analysis of GmCWINV4

The coding sequence of *GmCWINV4* without terminal codon was amplified and inserted into 35S:GFP vector, generating 35S:CWIN4‐GFP. The cell wall marker gene *AtEXPA1* (AT1G69530) and plasma membrane marker *AtPIP2A* (AT3G53420) were cloned into 35S:RFP vector, producing 35S:EXPA1‐RFP and 35S:PIP2A‐RFP, respectively. The primers used are listed in Table S7. Recombinant vectors were transformed into *Agrobacterium tumefaciens* strain GV3101. For subcellular localisation, 35S:CWIN4‐GFP with 35S:EXPA1‐RFP or 35S:PIP2A‐RFP were co‐infiltrated in 5‐week‐old *Nicotiana benthamiana* leaves and incubated for 3 days. For plasmolysis treatment, *N. benthamiana* leaves were treated with 15% (w/v) mannitol for 15 min. GFP and RFR signals were observed with a confocal microscope (FV‐1000, OLYMPUS).

*N. benthamiana* leaves infiltrated with 35S:CWIN4‐GFP or 35S:GFP (as a control) were sampled for enzyme activity measurement using Solid‐Acid Invertase Activity Assay Kit (Solarbio Science & Technology Co. Ltd., Beijing, China). Briefly, crude cell wall protein extracts were isolated from tobacco leaves and incubated with sucrose as the substrate according to the manufacturer's instructions. The resulting reducing sugars reacted with 3,5‐dinitrosalicylic acid (DNSA) to form a brown‐red coloured amine compound, and the absorbance was measured at 510 nm. Three independent replicates were performed for each experiment.

### 4.6 RNA In Situ Hybridisation

Briefly, the CDS region of *GmCWINV4* was PCR amplified with the reverse primer containing a T7 RNA polymerase binding site at its 5′ end. PCR products were used as template to generate digoxigenin‐labelled RNA probes using T7 RNA polymerase digoxigenin‐UTP labelling kit (Roche, Germany) according to the manufacturer's protocol; probes were hydrolysed to an average length of 150 bp. Developing soybean seeds at S1–S2 were fixed in FAA solution (50% ethanol, 5% acetic acid, 3.7% formaldehyde), subsequently embedded in paraffin wax, and sectioned at 8 μm thickness. Samples were hybridised with probes, and the signals were visualised with an Olympus BX63 microscope using DIC imaging.

### 4.7 GmCWINV4 Overexpression in Soybean and Seed Traits Analysis

For *GmCWINV4* overexpression, the coding sequence was cloned and inserted into the downstream of soybean *Ubiquitin* promoter in the pCAMBIA2300 vector. Transgenic soybean plants were generated in the Williams 82 genetic background by the cotyledonary node‐*A. tumefaciens*‐mediated transformation method. Seed length, width, and thickness were measured using a vernier calliper with 20 replicates per overexpression line. Seeds weight was measured using an electronic balance with 5 replicates per overexpression line. Seed oil and protein contents were determined using GrainSense analyser (Grainsense Ltd., Oulu, FINLAND) with 8 biological replicates.

To quantify the contents of fructose, glucose, and sucrose in seeds, mature seeds from Williams 82 and *GmCWINV4*‐overexpressing lines cultivated under greenhouse conditions were harvested. The seeds were ground in liquid nitrogen, and a 100 mg aliquot was utilised for soluble sugar extraction. Extraction was performed with zinc acetate solution (0.1 mL) and potassium ferrocyanide solution (0.1 mL) for 40 min. Subsequently, the mixture was centrifuged at 12 000 × g for 5 min at 4°C, and the supernatant was collected for sugar analysis. An aliquot of 10 μL of the solution was injected into a high‐performance liquid chromatography (HPLC) system (Shimadzu LC‐10ADvp) equipped with an NH_2_ column (4.6 × 250 mm) maintained at 40°C. Chromatographic separation was achieved using an acetonitrile‐water mobile phase (70:30, v/v) at a flow rate of 1 mL/min. Target compounds were identified by comparing the retention times of sample peaks with those of standard solutions (fructose, glucose, and sucrose) under identical HPLC conditions. The concentration of each soluble sugar was determined based on a standard curve (a reference?). Besides, histological and cytological analysis for mature seeds from WT and overexpressing lines were performed according to our previously described method (Han et al. 2025). Cell number and size from the inner surface of the cotyledons were estimated using ImageJ software (a reference).

### 4.8 Yeast One‐Hybrid Assay

The 922‐bp promoter fragment of *GmCWINV4* was cloned and ligated into the pAbAi vector to construct the bait plasmid pAbAi‐proGmCWINV4. The linearised bait plasmid was subsequently transformed into the yeast strain Y1HGold and employed to screen a cDNA library derived from developing soybean seeds according to the manufacturer's instructions for the Matchmaker Gold Yeast One‐Hybrid Library Screening System (Clontech). Positive yeast colonies grown on the screening medium comprising SD/−Ura/−Leu supplemented with AbA (100 ng/mL) were isolated for PCR amplification and Sanger sequencing. The resulting sequences were subsequently aligned against the Williams82 genome (https://phytozome‐next.jgi.doe.gov). In the Y1H point‐to‐point assay, the coding sequences of *GmZH‐HD27/36* were cloned into the prey vector pGADT7. Subsequently, the prey plasmids were transformed into the yeast strain Y1HGold harbouring pAbAi‐proGmCWINV4, with the empty vector pGADT7 as a negative control. Colonies grown on SD/−Leu medium were then plated onto SD/−Ura/−Leu medium containing 100 ng/mL AbA to evaluate the binding of transcription factors to the GmCWINV4 promoter. The primers used here were listed in Table S7.

### 4.9 Dual‐Luciferase Reporter Assay

The various lengths of the *GmCWINV4* promoter regions were cloned and ligated upstream of the firefly luciferase (*LUC*) gene to generate the dual‐luciferase reporter vector, pCAMBIA3301‐LUC‐RLUC. The coding sequences of *GmZF‐HD27/36* were cloned into the 35S:RFP vector to generate effector constructs. All recombinant plasmids were introduced into *A. tumefaciens* strain GV3101. Effector and reporter strains were co‐infiltrated into tobacco leaves at a ratio of 4:1 (v/v). Following a three‐day incubation period, LUC and Renilla luciferase (REN) activities were quantified using a Dual Luciferase Reporter Assay Kit (Vazyme, China). For chemiluminescence imaging, leaf samples were treated with 0.3% luciferin, incubated in darkness for 3 min, and subsequently visualised using a CCD imaging system (Tanon, China).

To investigate the sucrose‐induced expression of *GmZF‐HD36*, the 2000‐bp promoter region was cloned and ligated upstream of the *LUC* gene to construct the reporter vector, designated as pCAMBIA3301‐GmZFHDpro‐LUC‐RLUC. Subsequently, the reporter vector was infiltrated into tobacco leaves. After 3 days, distinct leaf regions were treated with a range of sucrose or mannitol (as a control) concentrations. After a subsequent 6‐h incubation, luciferase activity was visualised using CCD‐based imaging. The primers used here were listed in Table S7.

### 4.10 Electrophoretic Mobility Shift Assay

The coding sequence of GmZF‐HD36 was cloned into the pCold‐TF‐His vector and transformed into *Escherichia coli* strain Rosetta (DE3). Recombinant ZF‐HD‐His protein was purified using Ni‐NTA magnetic beads (Yeasen, China). To identify GmZF‐HD36‐binding sites, biotin‐labelled probes (including mutant probes) were synthesised by Sangon Biotech (Shanghai). Unlabeled probes of the same sequence were used as competitors in competition assays. All probe sequences used in this experiment are listed in Table S7. The purified protein was incubated with double‐stranded probes at room temperature for 30 min. The reaction mixture was electrophoresed on a non‐denaturing 6% polyacrylamide gel in 0.5 × TBE buffer at 60 V for 1 h at 4°C, followed by transfer to Biodyne B nylon membranes (Pall Corporation).

### 4.11 Sugar Treatment of Developing Seeds In Vitro

Immature soybean pods were harvested and surface‐sterilised by immersion in a 0.1% sodium dichloroisocyanurate (NADCC) solution for 10 min. After that, the pods were thoroughly rinsed multiple times with sterile distilled water, and seeds at the S1–S2 stage were carefully excised under a laminar flow hood. These seeds were then transferred into liquid Murashige and Skoog (MS) medium supplemented with 0%, 1%, and 3% (w/v) sucrose. Each treatment contained approximately 15 seeds and was incubated at 25°C for 8 h. There were three biological replicates for each treatment. Finally, all samples were rapidly frozen in liquid nitrogen and stored at −80°C.

### 4.12 Quantitative Real‐Time PCR (qRT‐PCR)

For the expression of GmCWINV4, we collected the developing seeds in different developmental stages (S1–S6) from Williams 82 and wild soybean W05 (*Glyma soja*). For sucrose‐induced expression of GmZF‐HD27/36, developing seeds cultured in liquid MS medium in vitro with exogenous sucrose were collected. The total RNA was extracted using the Trizol method and was reversely transcribed into cDNAs using the PrimeScript RT reagent Kit (TaKaRa, Dalian, China). qRT‐PCR was performed in the Bio‐Rad CFX Manager system (BIO‐RAD, Berkeley, CA, USA) with three biological replicates. The *GmTubulin* (Glyma.05G157300) was used as an internal control. All primers used here were listed in Table S7.

## Author Contributions

Wei Xu, Tianquan Yang, Tingshuang Yi conceived and designed the research. Di Wu, Yiran Ding and Tianquan Yang performed the experiments. Wei Xu and Tianquan Yang performed the data analyses. Di Wu and Yiran Ding conducted the transgenic experiments. Yang Tao, Xiaoyi Fu, Bing Han, and Juan Song cultivated all plant materials and analysed the phenotype. Wei Xu and Di Wu wrote the manuscript. Wei Xu, Tianquan Yang, Tingshuang Yi revised the manuscript. All authors read and approved the final manuscript.

## Funding

This work was supported by Major Program for Basic Research Project of Yunnan Province (Grant 202401BC070001), Yunnan Fundamental Research Projects (Grant 202301AT070561) and Yunnan Revitalisation Talent Support Program: Yunling Scholar Project (Grant XDYC‐YLXZ‐2024‐0021).

## Conflicts of Interest

The authors declare no conflicts of interest.

## Supporting information


**Figure S1:** Histological structure of early developing seed at globular (S1), heart (S2) and cotyledon (S3) stage.
**Figure S2:** Number of expressed genes and unique molecular identifiers (UMI).
**Figure S3:** The number of expressed genes and cluster‐specific genes in each seed subregion across three developmental stages (S1–S3).
**Figure S4:** Validation of *GmCWINV4* expression in seed subregions at globular stage and tissues expression patterns.
**Figure S5:** Subcellular localisation of plasma membrane and cell wall markers in tobacco epidermal cells.
**Figure S6:** Hydrolytic activity of GmCWINv4 on sucrose in CK, empty vector, GmCWINV5‐overexpressing line in tobacco leaves using 3,5‐dinitrosalicylic acid (DNSA) method.
**Figure S7:** Expression verification of GmCWINV4 overexpression lines.
**Figure S8:** Paraffin section observations of cell size and density in mature seeds from overexpression lines (OE2 and OE5) compared to the wild‐type Wm82.
**Figure S9:** The haplotype analysis of *GmCWINV4*.
**Figure S10:** Spatial expression of *GmZF‐HD36* and *GmZF‐HD27* in different seed clusters.
**Figure S11:** Relative luciferase activity for a series of 5′ truncated *GmCWINV4* promoter fragments and an internal deletion construct co‐expressed with GmZF‐HD36::RFP.
**Figure S12:** Transactivation activity of GmZF‐HD27 toward *GmCWINV4* via dual luciferase assay.
**Figure S13:** Response of GmZF‐HD27 and GmZF‐HD36 to sucrose treatment.

**Table S1:** Gene expression of soybean seed subregions at globular (S1), heart (S2) and cotyledon stages (S3).
**Table S2:** The cluster‐specific genes in each seed subregion across three developmental stages (S1–S3).
**Table S3:** Marker genes of each soybean subregion identified in this study and previous studies.
**Table S4:** Identification of sucrose transport‐ and catabolism‐genes in soybean genome.
**Table S5:** Expression level of sugar‐related genes within seed subregions across three developmental stages (S1‐S3).
**Table S6:** Candidate genes identified from positive clones obtained via yeast one‐hybrid (Y1H) screening.
**Table S7:** Primer and probe sequences used in this study.

## Data Availability

The clean data of spatial transcriptome sequencing reported in this paper has been uploaded to China National Center for Bioinformation (https://www.cncb.ac.cn/), with genome sequence archive number GSA: CRA044164.
