# Supplementary material for: Spatiotemporal Transcriptomics Reveals That a ZF‐HD–GmCWINV4 Module Controls Carbon Partitioning and Seed Size in Soybean
Source: Plant Biotechnol J. 2026 Oct 28:10.1111/pbi.70776. Online ahead of print. doi: 10.1111/pbi.70776 (PMC13714592; doi:10.1111/pbi.70776)
Supplement: Supplementary file 1 [file PBI-9999-0-s001.docx]

**Figure S1**. **Histological structure of early developing seed at globular (S1), heart (S2) and cotyledon (S3) stage.** The known subregions of soybean seeds were delineated.

**Figure S2**. **Number of expressed genes and unique molecular identifiers (UMI).**

(a) The average number of expressed genes per spot and the percentage of gene in mitochondrial (mt) and chloroplast (cp) genome.

(b) Pearson correlation of number between genes and UMI.

**Figure S3**. **The number of expressed genes and cluster‑specific genes in each seed subregion across three developmental stages (S1–S3)**.

**Figure S4. Validation of *GmCWINV4* expression in seed subregions at globular stage and tissues expression patterns.**

**(a)** The UMAP plot of spatial transcriptomic data showing distinct cell types/clusters in soybean seed at globular stage (Zhang et al., 2025, https://soybean-atlas.com/).

**(b)** The UMAP plot showing specific and high expression of *GmCWINV4* in the seed coat phloem (SC phloem.2) as inficated by red arrow.

**(c)** Tissue expression profile of *GmCWINV4* from public transcriptome databases (https://ngdc.cncb.ac.cn/soyomics). The gene shows preferential expression in reproductive organ (e.g. flower and seed).

**Figure S5.** **Subcellular localization of plasma membrane and cell wall markers in tobacco epidermal cells.** Tobacco (*Nicotiana benthamiana*) leaves were subjected to co-transformation utilizing 35S:GFP as an empty vector, in combination with either the plasma membrane marker AtPIP2A-RFP (upper panel) or the cell wall-associated protein AtEXPA1-RFP (lower panel). GFP: fluorescence (green), RFP: fluorescence (red), chlorophyll autofluorescence (magenta), BF: bright field. Scale bar: 50 μm.

**Figure S6. Hydrolytic activity of GmCWINV4 on sucrose in CK, empty vector, GmCWINV5-overexpressing line in tobacco leaves using 3,5-dinitrosalicylic acid (DNSA) method.**

**Figure S7. Expression verification of *GmCWINV4* overexpression lines.**

(a) RT-PCR detection of *GmCWINV4* in leaves of wild-type Wm82, OE2 and OE5; *GmTUB* served as an internal control. M: DNA marker.

(b) qRT-PCR quantification of *GmCWINV4* relative expression in leaves of wild-type Wm82, OE2 and OE5. Values are mean ± SD of three biological replicates. Student’s t-test, *** *P* < 0.001 compared with Wm82.

**Figure S8. Paraffin section observations of cell size and density in mature seeds from overexpression lines (OE2 and OE5) compared to the wild-type Wm82.** The upper panels depict the complete seed sections, whereas the lower panels provide magnified views of the cotyledon regions demarcated by red rectangles. Scale bars are included within each image.

**Figure S9. The haplotype analysis of *GmCWINV4*.**

(a) **Haplotype variation within the 2‑kb promoter and genic regions of** GmCWINV4**.** Haplotypes were defined based on SNP and INDEL variants extracted from 4,414 resequenced soybean accessions. Variant data were retrieved from the SoyMD public database ([https://yanglab.hzau.edu.cn/SoyMD/#/](https://yanglab.hzau.edu.cn/SoyMD/#/)).

(b) Media-joining network illustrating the genetic relatedness among 11 *GmCWINV4* haplotypes. Each circle corresponds to one distinct haplotype. Circle color denotes germplasm type: red for wild soybeans (*Glycine soja*), yellow for landraces, and purple for improved cultivars (*Glycine max*). Circle size is proportional to the number of accessions carrying each haplotype.

(c) Haplotype distribution of *GmCWINV4* across wild, landrace, and cultivated soybeans.

(d) Seed oil and protein contents associated with four *GmCWINV4* haplotypes. Significant differences are indicated by distinct lowercase letters (a–c; Tukey’s HSD, *P* < 0.05).

**Figure S10.** **Spatial expression of *GmZF‑HD36* and *GmZF‑HD27* in different seed clusters.** Circle size corresponds to the percentage of SupSpots within each cluster with detectable transcripts (UMI > 0). Circle color represents the average normalized expression level of the gene. *GmZF‑HD36* exhibits the high expression in cluster 0 and cluster 8 (seed coat phloem), indicating co-expression of *GmZF‑HD36* and *GmCWIN4*.

**Figure S11.** Relative luciferase activity for a series of 5′ truncated *GmCWINV4* promoter fragments (−922 bp, −615 bp, −532 bp, −154 bp) and an internal deletion construct (−922 to −201 bp) co-expressed with GmZF-HD36::RFP. The RFP empty vector together with the −922 bp promoter fragment served as the negative control. Data are presented as mean ± SD of three biological replicates. Student’s t-test, *** *P* < 0.001 compared with RFP; ns, no significant difference.

**Figure S12. Transactivation activity of GmZF-HD27 toward *GmCWINV4* via dual luciferase assay**.

(a) Dual-luciferase assay showing GmZF-HD27-mediated transcriptional activation of sequentially truncated *GmCWINV4* promoters (−922 bp, −615 bp, −532 bp, −154 bp) and an internal deletion construct (−922 bp to −201 bp).

Relative luciferase activity was measured for a series of 5′ truncated *GmCWINV4* promoter fragments co-expressed with GmZF-HD27::RFP. The RFP empty vector together with the −922 bp promoter fragment served as the negative control. Data are presented as mean ± SD of three biological replicates. Student’s t-test, *** *P* < 0.001 and * P < 0.05 compared with RFP; ns, no significant difference.

(b) Electrophoretic mobility shift assay (EMSA) of his-GmZF-HD27 fused protein binding to biotin probes with corresponding motifs and mutant sequences (M1). -, the absence of corresponding probes; +, the presence of corresponding probes; competitor: corresponding motif sequences without biotin.

**Figure S13. Response of** **GmZF-HD27 and GmZF-HD36 to sucrose treatment.**

**(a)** Relative expression of GmZF-HD27 and GmZF-HD36 under control (CK, no sucrose addition), 1% and 3% sucrose treatment in seeds at S1 stage (mean±SD, n=3, Student’s t-test, ** *P* < 0.01, *** *P* < 0.001).

**(b-d)** Sucrose treatment inducing the expression of GmZF-HD36 promoter in Tobacco leaves. The left half of tobacco leaves was transfected with *proGmZF-HD36*::LUC, whereas the right half was transfected with an empty LUC vector as a control. The leaves without sucrose and mannitol were used as negative control (**b**) or treated with 1% and 3% mannitol (Man) were used for osmotic stress control (**c**), and treated with 1% and 3% sucrose (Suc) were used for experiments (**d**). Luminescence intensity is indicated by the color scale. Man: mannitol; Suc: sucrose.

(**e**) Relative luciferase activity of *proGmZF-HD36*::LUC under mannitol and sucrose treatments. Values are mean ± SD of three biological replicates. Student’s t-test, ** *P* < 0.01; ns, not significant.
